# DC-Based Vaccines for Cancer Immunotherapy

**DOI:** 10.3390/vaccines8040706

**Published:** 2020-11-26

**Authors:** Chunmei Fu, Li Zhou, Qing-Sheng Mi, Aimin Jiang

**Affiliations:** 1 Center for Cutaneous Biology and Immunology, Department of Dermatology, Henry Ford Health System, Detroit, MI 48202, USA; cfu1@hfhs.org (C.F.); LZHOU1@hfhs.org (L.Z.); QMI1@hfhs.org (Q.-S.M.); 2 Immunology Program, Henry Ford Cancer Institute, Henry Ford Health System, Detroit, MI 48202, USA

**Keywords:** dendritic cells, vaccines, plasmacytoid DCs, exosomes, DC-targeted vaccines

## Abstract

As the sentinels of the immune system, dendritic cells (DCs) play a critical role in initiating and regulating antigen-specific immune responses. Cross-priming, a process that DCs activate CD8 T cells by cross-presenting exogenous antigens onto their MHCI (Major Histocompatibility Complex class I), plays a critical role in mediating CD8 T cell immunity as well as tolerance. Current DC vaccines have remained largely unsuccessful despite their ability to potentiate both effector and memory CD8 T cell responses. There are two major hurdles for the success of DC-based vaccines: tumor-mediated immunosuppression and the functional limitation of the commonly used monocyte-derived dendritic cells (MoDCs). Due to their resistance to tumor-mediated suppression as inert vesicles, DC-derived exosomes (DCexos) have garnered much interest as cell-free therapeutic agents. However, current DCexo clinical trials have shown limited clinical benefits and failed to generate antigen-specific T cell responses. Another exciting development is the use of naturally circulating DCs instead of in vitro cultured DCs, as clinical trials with both human blood cDC2s (type 2 conventional DCs) and plasmacytoid DCs (pDCs) have shown promising results. pDC vaccines were particularly encouraging, especially in light of promising data from a recent clinical trial using a human pDC cell line, despite pDCs being considered tolerogenic and playing a suppressive role in tumors. However, how pDCs generate anti-tumor CD8 T cell immunity remains poorly understood, thus hindering their clinical advance. Using a pDC-targeted vaccine model, we have recently reported that while pDC-targeted vaccines led to strong cross-priming and durable CD8 T cell immunity, cross-presenting pDCs required cDCs to achieve cross-priming in vivo by transferring antigens to cDCs. Antigen transfer from pDCs to bystander cDCs was mediated by pDC-derived exosomes (pDCexos), which similarly required cDCs for cross-priming of antigen-specific CD8 T cells. pDCexos thus represent a new addition in our arsenal of DC-based cancer vaccines that would potentially combine the advantage of pDCs and DCexos.

## 1. Introduction

As the professional antigen presenting cells (APCs), dendritic cells (DCs) play a critical role in the initiation and regulation of innate and adaptive immune responses, and have the unique ability to activate (prime) both naïve CD4 and CD8 T cells [1]. Cross-priming, a process in which DCs activate CD8 T cells by cross-presenting exogenous antigens onto MHC class I molecules [2,3], plays a critical role in generating CD8 T cell immunity against cancers and viruses, upon vaccination, as well as in the induction of CD8 T cell tolerance (cross-tolerance) [4,5,6,7]. Exploiting their ability to potentiate host effector and memory CD8 T cell responses critical for anti-tumor immunity, DC vaccines have emerged as one of the leading strategies for cancer immunotherapy [8,9,10,11]. Of note, vaccines with other APCs including B cells and macrophages have also been shown to generate T cell-mediated anti-tumor immunity [12]. Indeed, B cell vaccines represent an attractive alternative to DC vaccines, as B cell function in T cell activation has been shown to be resistant to immunosuppressive cytokines including IL-10, TGF-β and VEGF often present in the tumor microenvironment [12,13]. However, vaccines with these other APCs are under-studied, and DCs remain the overwhelming cell of choice for cell-based vaccines for cancer immunotherapy [14]. DCs comprise heterogenous populations including conventional DCs (cDCs), plasmacytoid DCs (pDCs) and monocyte-derived DCs (MoDCs) [11,15,16]. DC vaccines, of which the vast majority employ monocyte-derived DCs generated in vitro, are largely unsuccessful, only achieving objective immune responses in 5–15% of patients. Sipuleucel-T, which comprise blood cells enriched for antigen-presenting cells (APCs) including DCs, remains the only FDA (Food and Drug Administration)-approved “DC” cancer vaccine in over 10 years [17]. Despite largely disappointing clinical trials, the promising results from DC vaccine clinical trials using neoantigens offer an exciting new development on DC vaccines for cancer immunotherapies [18,19,20]. Recent discovery on the critical role of cDC1s (type 1 conventional DCs) in cross-priming tumor antigen-specific CD8 T cells and in determining the efficacy of cancer immunotherapies [21,22,23,24,25], further highlighted the importance of the development and refinement of DC-based vaccines either as monotherapy or combinational immunotherapies. 

There are two major hurdles of the success of DC vaccines: tumor-mediated immunosuppression and the functional limitations of the commonly used in vitro differentiated DCs [10,11]. As inert vesicles, DC-derived exosomes (DCexos) are resistant to regulation by tumor-related factors compared to DCs. Therefore, vaccines with DCexos might represent a new type of DC-based vaccines that could overcome tumor-mediated immunosuppression [26]. In vivo DC-targeted vaccines and the use of naturally circulating blood DCs also offer promising alternatives to in vitro-differentiated DCs used in the majority of clinical trials [27]. The promising clinical trials of pDCs, including a recent clinical trial using a human pDC cell line, and the potential of combining pDCs with cDCs, support further development of pDC-based cancer vaccines immunity [28,29,30]. The generation of previously unreported pDC-derived exosomes (pDCexos) [31] offer an exciting new addition in the arsenal of DC-based vaccines, as vaccines with pDCexos have the potential to combine the advantages of both pDC and DCexo vaccines.

## 2. In Vivo DC-Targeted Vaccines

Another major contributor to the lack of clinical benefits by DC vaccines is the functional limitation of ex vivo differentiated DCs, which were used in the vast majority of all DC-based clinical trials [32]. These DCs, which are differentiated from hematopoietic precursors, have been shown to exhibit less stimulating and functional capacity than naturally circulating DC subpopulations, specifically in their capacity to cross-present antigens to prime antigen-specific CD8 T cells [10,11]. To overcome this obstacle, one approach is delivering antigens to DCs in situ with antibodies targeting DC-specific receptors including DEC-205, Clec9A, Mannose Receptor (MR), DCIR2, DC-SIGN, Dectin-1, Siglec-H and Bst-2 [33,34,35]. In pre-clinical mouse models, DC-targeted anti-DEC-205 coupled with antigens have been shown to result in significantly increased efficiency of antigen presentation onto both MHCI and MHCII (MHC class II), with >1000 times cross-presentation compared to non-coupled protein antigens [36,37,38]. However, it should be noted that adjuvants are required, otherwise these antibodies coupled with antigens will lead to T cell tolerance instead of immunity [36,37]. As In vivo DC-targeted vaccines do not require the labor- and cost-intensive procedures of ex vivo DC differentiation and culturing, they offer an economical alternative that could be used for large-scale vaccinations [34,39,40]. 

Several clinical trials using DC-targeted antigens have shown that in vivo DC-targeted vaccines are safe with varied effects on immune responses [41,42,43]. In one phase I clinical trial, human anti-DEC-205 monoclonal antibody fused with tumor antigen NY-ESO-1 with adjuvant poly-ICLC and/or resiquimond induced both humoral and NY-ESO-1-specific T (CD4 and CD8) cell responses and led to partial clinical responses with no signs of toxicity [42]. More encouragingly, data from the combination of DC vaccines with anti-CTLA4 treatment indicated that four out of six patients showed a partial or complete response, which is higher than the 15% response rate observed for anti-CTLA4 monotherapy. Despite these promising results, clinical application was slowed by several challenges such as endogenous DCs in cancer patients being defective in their functional activity [15,44,45], specificity requirement of the targeted receptors on selected DC subsets and the limitation of one known tumor antigen at a time. For example, MR and DC-SIGN are targeted in two of the clinical trials, but whether these two receptors are specifically expressed on human DC subsets has been controversial [46]. Refinement of the specific targeting of antigens to DCs with nanoparticles or viral vectors, additional in situ mobilization and modulation of endogenous DCs using implantable or injectable biomaterial-based platforms, and the combining DC-targeted vaccines with immune checkpoint blockade (ICB, e.g., anti-CTLA4) and adoptive cell transfer will likely help advance the clinical application of DC-targeted vaccines [32,47] (Table 1). 

## 3. Naturally Circulating Primary DCs as Cancer Vaccines

To overcome the functional limitations of MoDCs commonly used in DC vaccines, another promising approach is to use freshly purified blood-derived primary DC subsets. Although rare in numbers, recent technological development of antibody-coated magnetic beads enables the isolation of sufficient numbers of naturally circulating DC subsets directly from peripheral for cancer vaccines [16,27,48]. Naturally circulating primary DCs could be readily activated/maturated and loaded with antigens, thus avoiding extensive culturing to preserve their functionality and prevent exhaustion. However, a direct comparison of the efficacy between naturally circulating DC subsets and in vitro cultured DCs has not been tested in clinical trials, therefore it remains unknown whether naturally circulating DCs are superior as cancer vaccines compared to in vitro cultured DCs [16,27]. Human naturally circulating DCs (nDCs) in the blood comprise two major types—pDCs and cDCs, that can be separated by different surface markers. cDCs can be further divided into CD1c (BDCA1)^+^ DCs (cDC2s), which are specialized in immune responses against bacterial and fungi, and CD141 (BDCA3)^+^ DCs (cDC1s), specialized in cross-presentation of tumor antigens to prime CD8 T cells [27]. 

Several clinical trials using naturally circulating DCs including cDC2s and/or pDCs have shown that nDC vaccines are safe and well-tolerated in patients, with the induction of antigen-specific immunity in some patients [28,49,50,51,52,53]. In the first clinical trial, naturally circulating DCs were first expanded with Flt3L to increase the yield of blood DCs from patients with melanoma, and strong immune responses were observed in several patients [49]. However, the purity of the isolated DCs was generally low and Flt3L treatment was later found to induce the expansion of regulatory T cells in melanoma patients [49,54], suggesting that the Flt3L treatment might not be a suitable clinical approach for nDC vaccines. Three phase I clinical trials using BDCA1^+^ cDC2s loaded with TAAs (tumor-associated antigens) for prostate cancer and melanoma have shown that they are safe and feasible [50,51,52]. Although immune responses or clinical effects were not observed in two of the clinical trials [50,52], the third clinical trial showed antigen-specific CD8 T cell responses correlated with improved progression-free survival (PFS) in 4 out of 14 patients [51]. Similarly, a clinical trial using isolated pDCs, which were activated with Fruhsommer-meningoencephalitis (FSME, tick-borne encephalitis) and loaded with three TAAs, induced tumor-specific CD8 T cell immunity correlated with improved progression-free survival (PFS) [28]. Based on these positive results, two clinical trials were carried out by the same group using cDC2s, pDCs or both cDC2s and pDCs for melanoma and castration-resistant prostate cancer (CRPC) patients [27]. To stimulate the DCs in the combination trials, DCs are activated with protamine/mRNA which can induce maturation of both pDCs and BDCA1^+^ mDCs [55]. Westdorp et al. have recently reported on one of the clinical trials, a phase IIa CRPC clinical trial with cDC2s and/or pDCs [29]. Not surprisingly, this phase IIa CRPC clinical trial has shown that vaccinations with cDC2s and/or pDCs are safe and lead to antigen-specific T cell responses in the majority of patients [29]. However, no significant difference was observed between different DC subset groups, and clinical efficacy of single DC subset vaccination versus the combination of pDCs and cDC2s will only be assessed in the follow-up phase II/III clinical trials [29]. Overall, vaccines with naturally circulating blood DCs including cDC2s and pDCs have shown encouraging data in clinical trials and represent a promising future direction for DC-based vaccines. However, CD141^+^ cDC1s, the critical player for priming anti-tumor CD8 T cells and generating CD8 T cell immunity, have not been used for cancer immunotherapy, likely due to their low numbers in peripheral blood [11]. The inability to use clinically-relevant CD141^+^ cDC1 subsets and the low yield of high purity DC subsets in peripheral blood remain the major hurdles for using nDCs for cancer immunotherapy (Table 1). 

## 4. Plasmacytoid DC-Based Cancer Vaccines

Although initially regarded as the less efficient antigen presenting cells (APCs), pDCs can present antigens to activate both CD4 T cells and CD8 T cells through cross-presentation [56,57]. pDCs are generally thought to be tolerogenic and play a suppressive role in tumors, as the accumulation of pDCs in multiple tumors including melanoma, head and neck, breast, and ovarian cancers has been associated with poor prognosis [58,59,60,61,62]. On the other hand, activation of pDCs has also been reported to induce immunogenic responses and shown therapeutic efficacy in human cancers, indicating pDC-mediated anti-tumor immunity [27,28,29,45,59,63]. However, initial studies comparing vaccines between cDCs and pDCs seem to suggest the cDC vaccines achieve better anti-tumor efficacy compared to pDC vaccines. Using a malignant glioma mouse model, Dey et al. have shown that cDCs induced stronger anti-tumor CD8 T cell responses and better anti-tumor efficacy compared to pDCs [64]. Using human pDCs and cDC2s differentiated from CD34^+^ hematopoietic stem cells, it has been reported that BDCA1^+^ cDC2s are superior in generating antigen-specific CD8 T cell immunity, although pDCs are superior in activating natural killer (NK)cells [65], suggesting that vaccines that combine both cDC2s and pDCs might achieve better anti-tumor efficacy. 

As we have mentioned in the previous section, clinical trials using naturally circulating pDCs induced tumor-specific CD8 T cell immunity correlated with improved progression-free survival (PFS) [28]. In the phase IIa CRPC clinical trial, no significant difference was observed between vaccinations with cDC2s versus pDCs [29], although whether combined vaccines using both cDC2s and pDCs achieve better efficacy remains to be assessed in follow-up clinical trials. However, accumulated evidence has emerged showing that both pDCs and cDCs are required to achieve optimal cross-priming and CD8 T cell immunity under diverse conditions [66,67,68,69,70,71]. Thus, employing multiple subsets of DCs might be a feasible approach to improve the anti-tumor efficacy of DC vaccines.

The most exciting development in pDC-based cancer vaccines comes from a recent phase I clinical trial (GeniusVac-Mel4) on melanoma patients using pDCs from an allogeneic pDC cell line [30]. This pDC cell line is derived from malignant leukemic cells of a patient with PDC leukemia which have been shown to function as a potent antigen-presenting cells in preclinical studies [72,73,74]. These pDC cells are loaded with four melanoma antigens and irradiated prior to administration to prevent further proliferation of pDCs in the patients. The pDC vaccine was well tolerated with no serious vaccine-induced side effects and induced strong priming of antigen-specific T cells with signs of clinical responses [30]. Strikingly, there was no allogeneic responses to the vaccines, suggesting the feasibility of using allogenic DCs as cancer vaccines [75]. Although vaccination with allogeneic DCs still requires an MHCI match (human leukocyte antigen (HLA)–A2.1) to enable antigen presentation, the use of the pDC cell line requires no demanding procedures on cancer patients and is amendable to manufacture standard in unlimited supply at low cost. Further studies are warrantied to test the suitability of pDC cell lines as off-the shelf replacement of in vitro differentiated DCs or naturally circulating DCs (Table 1).

## 5. DC-Derived Exosomes as Cancer Vaccines

Exosomes are small inert membrane vesicles about 30–150 nm diameter in sizes that form within late multivesicular endosomal compartments containing proteins, lipids and nucleic acids, and play an important role in intercellular communications and material transfer of their cargo [26,76,77]. DCs process exogenous antigens in endosomal compartments such as multivesicular endosomes which can fuse with plasma membrane to release exosomes (DCexos). DCexos express MHC class I and class II (often complexed with antigenic epitopes) as well as co-stimulatory molecules, and have been shown to prime antigen-specific CD8 T cells [78,79,80,81]. As inert vesicles, DCexos are more resistant to immunomodulation by tumors or tumor microenvironment (TME), have much longer half-life in vivo and can be stored for longer period of time compared to DCs [26]. Due to their resistance to tumor immunosuppression, bioavailability and biostability, DCexos have garnered much interest as cell-free therapeutic vaccines that are resistant to immunosuppression often observed in vaccine hosts. Indeed, DCexo vaccines exhibited better efficacy to eradicate tumors than DC vaccines in a T cell-dependent and MHC-restricted manner [82], thus supporting their clinical application as cancer vaccines [26,83,84,85]. Of note, whether pDCs generate exosomes and how they function in priming CD8 T cells have not been investigated, despite studies showing that pDC functions were regulated by exosomes [86,87,88].

Up to now, two phase I and one phase II clinical trials with DCexos have been concluded in cancer patients [89,90,91]. In the two phase I clinical trials, DCexos were obtained from autologous immature MoDCs and loaded with HLA-restricted Melanoma-Associated antigen (MAGE) peptide epitopes, and then infused into patients with advanced non-small cell lung cancer (NSCLC) and metastatic melanoma, respectively [89,90]. Although both DCexo vaccines have shown that DCexos are safe and well-tolerated in patients, they failed to induce strong antigen-specific T cell responses. In the NSCLC trial, no antigen (MAGE)-specific T cell responses were detected by in vitro assays on peripheral blood mononuclear cells (PBMCs), although increases in systemic immune responses against MAGE by delayed type hypersensitivity assay (DTH)were observed in some patients [89]. In the melanoma clinical trial, no MAGE-specific CD4 and CD8 T cell responses were detected, although T cell responses against MART1 which was not included in the vaccines were observed [90]. On the other hand, increased NK cell effector functions were observed in both clinical trials, thus suggesting that DCexos might have the capacity to induce NK cell functions in vivo, likely through a NKG2D-dependent mechanism [92]. As both clinical trials used DCexos generated from immature MoDCs, and previous studies have shown DCexos from mature DCs to be more potent in mediating T cell priming [93,94]; the lack of antigen-specific T cell responses was partially attributed to the use of immature MoDCs. Thus, in the phase II clinical trial for advanced NSCLC, patients were vaccinated with peptide-loaded DCexos from IFN-γ-matured MoDCs [91]. While this clinical trial again showed that DCexo vaccinations were safe and DCexos stimulated NK cell functions, these DCexos from IFN-γ-matured MoDCs also failed to induce antigen-specific T cell responses even with multiepitope loading using cyclophosphamide (CTX) as adjuvant. As all three clinical trials used peptide-loaded DCexos from autologous MoDCs, based on the presumed critical role of exosomal MHCI–antigen complexes in T cell priming, the lack of antigen-specific T cell responses suggest that exosomal MHC–antigen complexes on peptide-pulsed DCexos might not be critical and/or sufficient for priming T cells in vivo. Indeed, the Gabrielsson group has shown that while peptide-loaded DCexos failed to prime antigen-specific CD8 T cell, protein-loaded DCexos were able to prime antigen-specific CD8 T cells in vivo [95]. Follow-up studies by the same group have further shown that OVA protein-loaded DCexos induced strong anti-tumor CD8 T cell immunity independent of exosomal MHCI [96], suggesting that these DCexos did not rely on exosomal MHCI–antigen complexes for priming CD8 T cells. Similarly, DCexos derived from α-fetoprotein (AFP)-expressing DCs have been shown to induce allogeneic anti-tumor immunity in a hepatocellular carcinoma (HCC) mouse model [97]. These studies led to the proposal to develop allogeneic DCexos as impersonalized cancer vaccines without an MHC match between DCexo donors and vaccine recipients [98]. However, how DCexos prime allogeneic versus syngeneic CD8 T cell responses to generate anti-tumor immunity have not been well-studied and remain poorly understood. Given that current DCexo studies are focusing on peptide- or protein-loaded DCexos from conventional DCs (cDCs) [26,99], there is a critical need to expand our studies with new approaches to generate DCexos capable of priming CD8 T cells in vivo, and elucidate the underlying mechanisms of their functions in generating anti-tumor immunity (Table 1).

Although we have focused on DCexos as cancer vaccines, it should be noted that exosomes from other cells such as macrophages, NK cells, B cells, T cells, mesenchymal stem cells and tumor cells have also been employed as cancer vaccines [100,101]. Interestingly, tumor cell-derived exosomes (TEX) could be immunogenic and pro-inflammatory, although most of these exosomes appear to be immune suppressive [100,101]. As TEX contain a rich panel of tumor antigens that could be presented by APC to induce T and B cell responses, TEX vaccines have emerged as a promising new cell-free therapeutic agent in cancer immunotherapy [102]. In addition, exosomes could be engineered to modify surface molecules on exosomes to improve targeting of exosomal contents and induce tumor cell apoptosis, to modify exosomal contents to deliver cargos such as miRNA and cytokines for immune modulation, leading to improved anti-tumor efficacy [103,104].

## 6. Plasmacytoid DC-Derived Exosomes as Potential Cancer Vaccines

Although pDC-based vaccines have shown promising results in multiple clinical trials [28,29,30], how pDCs’ functions in generating anti-tumor CD8 T cell immunity versus promoting immune tolerance are regulated remains poorly understood. In fact, even the roles of pDCs in cross-priming CD8 T cells in vivo remain controversial, with a number of reports showing that pDCs did not play a role in cross-priming while other reports supporting that pDCs did cross-prime CD8 T cells in vivo [105,106,107,108,109,110,111,112,113,114,115]. Thus, better understanding whether and how pDCs cross-prime CD8 T cells to generate anti-tumor CD8 T cell immunity will be crucial to advance these promising pDC-based immunotherapies clinically.

**Figure 1:**
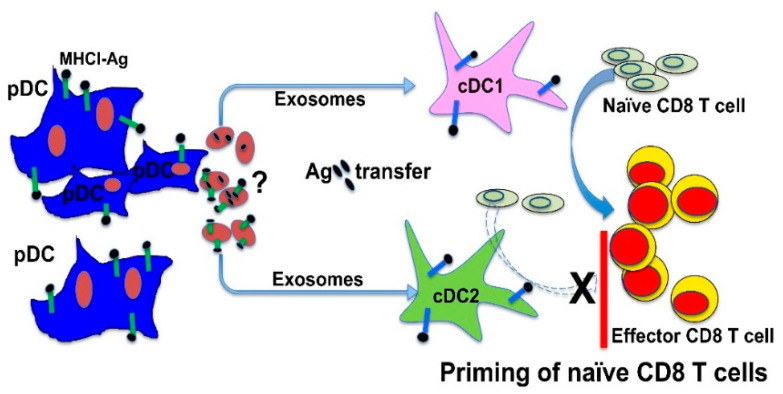
A novel mechanism for plasmacytoid DCs (pDCs) to cross-prime antigen-specific CD8 T cells by transferring antigens to bystander DCs through exosomes. Upon pDC-targeted vaccination, pDCs achieve cross-priming by transferring targeted antigens to conventional DCs (cDCs). Antigen transfer from pDCs to cDCs is mediated through pDC-derived exosomes (pDCexos), which similarly require bystander cDCs to prime antigen-specific CD8 T cells. While both cDC1s (type 1 cDCs) and cDC2s (type 2 cDCs) acquire antigens from pDCs similarly, cDC1s play a non-redundant role in pDC-mediated cross-priming, especially effector differentiation of antigen-specific CD8 T cells. How antigens are transferred from pDCexos to bystander cDCs remains unclear, and future studies will be required to determine whether pDCexos carry and transfer MHCI–Ag complexes to cDCs or carry intact antigens to be processed and presented by cDCs as reported for protein-loaded DCexos [95].

As both human and murine pDCs have been shown to be capable of cross-presentation in vitro [116,117,118,119,120], it seems that the priming of CD8 T cells instead of cross-presentation likely contribute to the opposite results regarding pDCs’ function in cross-priming in vivo. Previous studies have shown that immunization of pDC-targeted anti-Siglec-H and anti-Bst2 antibodies that express antigens of interest (anti-Siglec-H-Ag and anti-Bst2-Ag), specifically delivered antigens to pDCs but not cDCs in vivo [121,122]. As immunization with anti-Siglec-H-Ag has been shown to prime CD4 T cell to induce tolerance either with or without adjuvant [121], our group decided to first use pDC-targeting anti-Siglec-H-OVA to examine whether pDCs cross-prime CD8 T cells in vivo, and if so whether pDC-mediated cross-priming similarly leads to CD8 T cell tolerance. To our surprise, immunization with anti-Siglec-H-OVA plus CpG as adjuvant led to strong cross-priming of OVA-specific CD8 T cells and durable CD8 T cell immunity [31]. As CD4 T cell tolerance was observed upon immunization with anti-Siglec-H-OVA with or without adjuvants, our findings suggest that vaccination by anti-Siglec-H-OVA with adjuvants could simultaneously lead to CD4 T cell tolerance and CD8 T cell immunity; therefore, CD4 and CD8 T cell responses are likely regulated independently. More surprisingly, cross-presenting pDCs required cDCs to achieve cross-priming in vivo by transferring antigens to cDCs [31]. Firstly, depleting cDCs using CD11c-DTR chimeras led to substantially reduced cross-priming, suggesting that cDCs are required for in vivo cross-priming upon pDC-targeted vaccination. Secondly, when we isolated pDCs and cDCs after immunization, non-targeted cDCs instead of antigen-targeted pDCs were able to cross-prime naïve OVA-specific CD8 T cells, indicating that cross-presenting pDCs require the help of bystander cDCs to achieve cross-priming in vivo (Figure 1). Finally, we observed that the non-targeted cDCs also expressed the MHCI–OVA antigen (H–2K^b^–SIINFEKL) complexes, coupled with previous study showing that anti-Siglec-H antibodies were not detected in cDCs [31], suggesting that pDCs likely transfer antigens to non-targeted cDCs to achieve cross-priming. To confirm this conclusion on pDC-mediated cross-priming in vivo, we established an in vitro co-culture system where only pDCs had access to antigens. We found that, while cross-presenting pDCs were unable to prime antigen-specific CD8 T cells by themselves, adding naïve bystander cDCs to the pDCs/antigen-specific CD8 T cells co-cultures induced strong priming of antigen-specific CD8 T cells [31]. Our further studies showed that surface expression of MHCI–OVA antigen complexes on naïve bystander cDCs was detected after co-cultured with cross-presenting pDCs, suggesting that cross-presenting pDCs indeed transferred antigens to bystander cDCs. Taken together, our in vivo and in vitro data suggest that cross-presenting pDCs conferred naïve bystander cDCs the capability of cross-priming CD8 T cells by transferring antigens to cDCs [31] (Figure 1).

Among cDCs, Batf3-dependent cDC1s are recognized as the superior APCs in cross-presenting exogenous antigens including tumor antigens to prime CD8 T cells [123,124,125]; we thus asked whether cDC1s and cDC2s differed in their role in mediating pDCs’ function in cross-priming. Interestingly, cDC1s and cDC2s exhibited similar efficiency in acquiring antigens from cross-presenting pDCs. However, when cross-priming was examined, we observed that cDC1s played a critical role in pDC-mediated cross-priming, as cDCs lack of cDC1s failed to support pDC-mediated cross-priming in vitro. Consistent with these in vitro observations, Batf3^−/−^ mice (lack of both CD8^+^ and CD103^+^ cDC1s) exhibited significant reduced cross-priming, in particular effector differentiation of antigen-specific CD8 T cells upon pDC-targeted vaccinations [31]. Taken together, our data suggest that while both cDC1s and cDC2s were equally efficient in acquiring antigens from cross-presenting pDCs, cDC1s played a critical role in pDC-mediated cross-priming independent of their function in antigen presentation (Figure 1).

How do cross-presenting pDCs transfer antigens to bystander cDCs? Experiments using transwells and pDC supernatants demonstrated that antigen transfer from pDCs to cDCs was mediated by soluble factor(s). Further studies showed that antigen transfer from pDCs to bystander cDCs was mediated by previously unreported pDC-derived exosomes (pDCexos). Importantly, pDCexos primed naïve antigen-specific CD8 T cells only in the presence of bystander cDCs, similar to cross-presenting pDCs, thus identifying pDCexo-mediated antigen transfer to cDCs as a novel mechanism for pDCs to achieve cDC-dependent cross-priming. The pDCexo-mediated antigen transfer to cDCs is not limited to targeting pDCs via Siglec-H. Using pDC-targeted anti-Bst2-antigen [122], which we have shown to similarly require cDCs for cross-priming, we were able to show that pDCs cultured with anti-Bst2-antigen produced pDCexos, and these pDCexos similarly induced cross-priming only in the presence of bystander cDCs by transferring antigens to cDCs. Taken together, our data suggested that pDCs targeted with anti-Bst2-antigen and anti-Siglec-H-antigen shared the same mechanism of achieving CD8 T cell priming by transferring antigens to cDCs via pDCexo (Figure 1).

The identification of previously unreported pDCexos offers an exciting new addition to current DCexo collections. As a clinical trial of pDC vaccine using a human pDC cell line has shown promising results, pDCexo vaccines have the potential to combine the advantages of both DCexo and pDC vaccines (Table 1). As inert vesicles, pDCexos will be more resistant to cancer-mediated immunosuppression with superior biostability than pDCs. The availability of multiple well-characterized human pDC cell lines [126,127], including the one used the inGeniusVac-Mel4 clinical trial [30,126,127,128], will provide an unlimited supply of pDCexos at low cost without demanding procedures on vaccine patients. Further characterization of these newly identified pDCexos will be required to determine their potential application as cancer vaccines.

On the other hand, the identification of pDCexos also raises several interesting questions. As DC-targeted antigens such as anti-DEC-205 has been shown to be about 1000 times more efficient in cross-presentation than soluble protein antigens [37], it will be interesting to examine whether pDCexos generated with pDC-targeted antigens are similarly more efficient in cross-priming than pDCexos generated with non-targeted protein antigens. Another related question is how do pDCexos generated with pDC-targeted antigens transfer antigens to bystander cDCs: whether pDCexos carry MHCI–antigen (MHCI–Ag) complexes or intact antigens (conjugated with pDC-targeted antibodies) to be transferred to bystander cDCs (Figure 1). Previous studies have shown that intact OVA protein antigens were carried by DCexos generated with soluble OVA protein [129], whose uptake was likely mediated by receptor-mediated endocytosis similar to pDC-targeted antigens [130], thus strongly suggesting that pDCexos might also carry intact antigens to be transferred to bystander cDCs. As these OVA-loaded DCexos have been shown to induce strong allogeneic CD8 T cell immunity independent of exosomal MHCI [95,96], it is tempting to speculate that pDCexos generated with pDC-targeted protein antigens might similarly prime allogeneic antigen-specific CD8 T cells independent of exosomal MHCI. Further studies will be required to determine whether pDCexos could be developed as broadly applicable vaccines without MHCI match between pDCexo donors and vaccine recipients.

Our identification of pDCexos also raises the interesting question of whether cDCs loaded with cDC-targeted antigens similarly generate DCexos. If so, whether these cDCexos exhibit enhanced cross-priming capacity compared to cDCexos generated with non-targeted soluble protein antigens, and whether these cDCexos differ from pDCexos in their function in cross-priming. Further studies are required to further characterize these pDCexos and/or cDCexos generated with DC-targeted antigens to determine their potential as cancer vaccines.

## Figures and Tables

**Table 1:** Summary of current and potential dendritic cell (DC)-based cancer vaccines.

DC Vaccines	Advantages	Disadvantages
In vitro generated Monocyte-derived DCs (MoDCs)	Simple and tested differentiation protocols to obtain large number of MoDCs for vaccinations, easily adapted to different maturation stimuli and antigen loading approaches, safe and well-tolerated in patients in hundreds of clinical trials	MoDCs generated in vitro are functionally and genetically distinct from natural/primary DCs, long-term culture might lead to decreased migratory capacity and functionality loss, and objective clinical responses were only detected in 5–15% of patients
DCs generated in vitro from CD34^+^ hematopoietic precursors	DCs phenotypically similar to multiple primary DC subsets including Langerhans cells (LC), conventional DCs (cDCs) and plasmacytoid DCs (pDCs) could be generated	Heterogenous populations after differentiation, difficulty for standardization
DC-derived exosomes (DCexos)	As inert vesicles, more resistant to tumor-mediated suppression compared to DCs, superior bioavailability and biostability	Limited clinical benefits, unable to induce strong antigen-specific T cell responses, clinical trials limited to peptide-loaded DCexos from MoDCs
In vivo DC-targeted vaccines	Require no labor-intensive and costly DC preparation, could target subsets of DCs and substantially increase cross-presentation of targeted antigens, and are easily scalable	Difficulty to achieve specificity in vivo, the number of antigens targeted by antibodies could be limited and have to overcome defective endogenous DCs in cancer patients
Naturally circulating blood DCs (nDCs)	Both cDC2s and pDCs have been shown to induce antigen-specific T cell responses correlated with improved PFS, rapid and standardized production of nDC vaccines, and potential for synergy by combining multiple subsets of pDCs and cDCs.	Immune responses were not detected in some clinical trials using cDC2s, inability to isolate sufficient BDCA3^+^ cDC1s, limitation on DC numbers and dysfunctional DCs from cancer patients.
Allogeneic pDC cell line	No demanding procedures on vaccine patients, ready-to-use, unlimited supply for easy scaling-up and low cost.	Still require MHC match for antigen presentation, potentially detrimental allogeneic responses, and need more clinical data for assessment.
pDC-derived exosomes (pDCexos) as potential cancer vaccines	As inert vesicles, more resistant to tumor-mediated suppression compared to DCs, superior biostability, and the potential of using pDC cell lines further improves bioavailability and lowers cost	pDCexos were only reported recently, further characterization of these pDCexos and pre-clinical and clinical data are required to assess their potential as cancer vaccines
